# Supplementary material for: Influence of Climate and Local Habitat Characteristics on Carabid Beetle Abundance and Diversity in Northern Chinese Steppes
Source: Insects. 2019 Dec 24;11(1):19. doi: 10.3390/insects11010019 (PMC7023069; doi:10.3390/insects11010019)
Supplement: Supplementary file 1 [file insects-11-00019-s001.pdf]

Influence of climate and local habitat characteristics on carabid beetle abundance and diversity in northern Chinese steppes

Noelline Tsafack [1,*], Yingzhong Xie [1], Xinpu Wang [1,*] and Simone Fattorini [2]

[1] School of Agriculture, Ningxia University, 489 Helanshan West Road, 750021 Yinchuan, China; xieyz@nxu.edu.cn
[2] Department of Life, Health, and Environmental Sciences, University of L'Aquila, 67100 L'Aquila, Italy; simone.fattorini@univaq.it
* Correspondence: noelline.tsafack@gmail.com (N.T.); wangxinpu@nxu.edu.cn (X.W.)

Supplementary Material

Table S1. Variance inflation factors (VIF) of environmental variables used in the random effect eigenvector spatial filtering (RE-ESF).

		Grassland Type			
		**Desert Steppe**	**Desert Steppe without ST**	**Typical Steppe**	**Meadow Steppe**
Vegetation	PB	1.52	1.26	3.03	1.95
	PC	2.20	2.19	2.83	1.64
	PD	4.62	4.38	1.83	1.71
	PH	2.16	2.09	3.50	2.98
	PSD	2.91	2.90	1.67	1.55
Soil	SBD	1.52	1.48	1.49	2.21
	SL	1.58	1.57	3.63	1.34
	SM	1.65	1.46	1.46	1.92
	ST	10.65	------	1.63	3.53
Climate	Hum	2.85	2.73	4.00	2.91
	Prec	9.05	2.89	2.20	2.96
	Temp	4.14	3.45	2.89	1.92

Desert steppe without ST: VIF values after removing variable ST (Because VIF > 10). Predictors abbreviations: PB: Plant dry biomass, PC: Plant cover, PD: Plant density, PH: Plant height, PSD: Plant species diversity (richness); SBD: Soil bulk density, SL: Soil litter, SM: Soil moisture, ST: Soil temperature; Hum: Humidity, Prec: Precipitation, Temp: Temperature.

Table S2. Results of RE-ESF analysis (Random Effect Eigenvector Spatial Filtering) between habitat characteristics and carabid richness (Margalef index) for the three grassland types.

**Variables**		**Grassland Type**		
		**Desert Steppe**	**Typical Steppe**	**Meadow Steppe**
**r^2**		0.40	0.13	0.23
Vegetation	PB	0.06 ± 0.07 (0.346)	0.04 ± 0.06 (0.545)	-0.11 ± 0.07 (0.104)
	PC	0.05 ± 0.09 (0.597)	0.09 ± 0.06 (0.131)	-0.04 ± 0.06 (0.580)
	PD	−0.20 ± 0.12 (0.122)	0.06 ± 0.05 (0.214)	0.04 ± 0.07 (0.523)
	PH	−0.03 ± 0.09 (0.772)	−0.00 ± 0.07 (0.971)	0.06 ± 0.10 (0.503)
	PSD	−0.20 ± 0.10 (0.061)	−0.01 ± 0.05 (0.836)	0.02 ± 0.06 (0.804)
Soil	SBD	0.07 ± 0.07 (0.360)	−0.07 ± 0.04 (0.113)	−0.08 ± 0.07 (0.306)
	SL	**0.16 ± 0.08 (0.044)**	−0.06 ± 0.07 (0.411)	0.07 ± 0.06 (0.283)
	SM	0.15 ± 0.07 (0.056)	−0.03 ± 0.04 (0.429)	0.04 ± 0.07 (0.583)
	ST	**----**	−0.02 ± 0.05 (0.643)	0.13 ± 0.10 (0.189)
Climate	Hum	**0.26 ± 0.10 (0.016)**	**0.22 ± 0.07 (0.002)**	−0.05 ± 0.09 (0.601)
	Prec	**−0.33 ± 0.10 (0.003)**	−0.02 ± 0.05 (0.762)	0.10 ± 0.09 (0.246)
	Temp	0.21 ± 0.12 (0.079)	**0.17 ± 0.06 (0.007)**	**0.17 ± 0.07 (0.018)**
	Intercept	**0.37 ± 0.06 (<0.001)**	**0.97 ± 0.04 (<0.001)**	**1.10 ± 0.05 (<0.001)**

r^2 = adjusted coefficient of determination. Parameter estimated coefficients (± standard error) and *p*-values (in parentheses) are given for each predictor. Significant effects are in bold. Predictors abbreviations as in Table S1.

Table S3. Results of RE-ESF analysis between habitat characteristics and carabid diversity (Shannon-Wiener index) for the three grassland types.

**Variables**		**Grassland Type**		
		**Desert Steppe**	**Typical Steppe**	**Meadow Steppe**
**r²**		0.34	0.29	0.34
Vegetation	PB	0.07 ± 0.06 (0.219)	0.04 ± 0.05 (0.412)	**-0.10 ± 0.05 (0.033)**
	PC	0.05 ± 0.08 (0.504)	**0.11 ± 0.05 (0.016)**	-0.01 ± 0.04 (0.746)
	PD	−0.21 ± 0.11 (0.059)	**0.08 ± 0.04 (0.026)**	0.06 ± 0.05 (0.179)
	PH	−0.02 ± 0.08 (0.811)	0.00 ± 0.05 (0.938)	−0.00 ± 0.06 (0.995)
	PSD	−0.17 ± 0.09 (0.063)	−0.03 ± 0.03 (0.406)	0.02 ± 0.04 (0.612)
Soil	SBD	0.01 ± 0.06 (0.891)	−0.05 ± 0.03 (0.147)	−0.02 ± 0.05 (0.752)
	SL	**0.16 ± 0.06 (0.022)**	−0.06 ± 0.05 (0.272)	0.02 ± 0.04 (0.544)
	SM	0.12 ± 0.06 (0.069)	−0.02 ± 0.03 (0.595)	0.01 ± 0.05 (0.792)
	ST	**----**	−0.04 ± 0.03 (0.251)	0.11 ± 0.06(0.096)
Climate	Hum	**0.22 ± 0.09 (0.014)**	**0.21 ± 0.05 (<0.001)**	−0.03 ± 0.06 (0.624)
	Prec	**−0.31 ± 0.09 (0.001)**	0.01 ± 0.04 (0.807)	0.11 ± 0.06 (0.058)
	Temp	**0.20 ± 0.10(0.046)**	**0.18 ± 0.05 (<0.001)**	**0.18 ± 0.05 (<0.001)**
	Intercept	**0.31 ± 0.05 (<0.001)**	**0.85 ± 0.03 (<0.001)**	**0.91 ± 0.03 (<0.001)**

r^2 = adjusted coefficient of determination. Parameter estimated coefficients (± standard error) and *p*-values (in parentheses) are given for each predictor. Significant effects are in bold. Predictors abbreviations as in Table S1.

Table S4. Results of RE-ESF analysis between habitat characteristics and carabid diversity (exponential Shannon-Wiener index) for the three grassland types.

**Variables**		**Grassland Type**		
		**Desert Steppe**	**Typical Steppe**	**Meadow Steppe**
$\mathbf{r^2}$		0.35	0.27	0.33
Vegetation	PB	0.12 ± 0.12 (0.319)	0.08 ± 0.12 (0.505)	−0.15 ± 0.13 (0.250)
	PC	0.07 ± 0.16 (0.677)	**0.26 ± 0.11 (0.021)**	−0.07 ± 0.12 (0.537)
	PD	−0.37 ± 0.22 (0.102)	**0.20 ± 0.09 (0.023)**	0.17 ± 0.12 (0.157)
	PH	−0.10 ± 0.17 (0.554)	−0.01 ± 0.12 (0.953)	0.07 ± 0.18 (0.697)
	PSD	−0.36 ± 0.18 (0.058)	−0.06 ± 0.09 (0.450)	0.05 ± 0.12 (0.679)
Soil	SBD	−0.00 ± 0.13 (0.986)	−0.15 ± 0.08 (0.065)	−0.10 ± 0.14 (0.497)
	SL	**0.31 ± 0.13 (0.026)**	−0.12 ± 0.13 (0.337)	0.05 ± 0.12 (0.684)
	SM	0.26 ± 0.13 (0.059)	−0.04 ± 0.08 (0.643)	0.10 ± 0.13 (0.459)
	ST	**----**	0.15 ± 0.08 (0.081)	0.28 ± 0.18 (0.118)
Climate	Hum	**0.51 ± 0.18 (0.010)**	**0.46 ± 0.13 (<0.001)**	−0.10 ± 0.17 (0.573)
	Prec	**−0.61 ± 0.18 (0.002)**	0.07 ± 0.10 (0.480)	0.18 ± 0.16 (0.255)
	Temp	**0.44 ± 0.21 (0.041)**	**0.40 ± 0.11 (<0.001)**	**0.54 ± 0.13 (<0.001)**
	Intercept	**1.52 ± 0.10 (<0.001)**	**2.59 ± 0.07 (<0.001)**	**2.78 ± 0.09 (<0.001)**

r^2 = adjusted coefficient of determination. Parameter estimated coefficients (± standard error) and *p*-values (in parentheses) are given for each predictor. Significant effects are in bold. Predictors abbreviations as in Table S1.

Table S5. Results of RE-ESF analysis between habitat characteristics and carabid dominance (Berger-Parker index) for the three grassland types separately.

**Variables**		**Grassland Type**		
		**Desert Steppe**	**Typical Steppe**	**Meadow Steppe**
**r^2**		0.26	0.15	0.22
Vegetation	PB	−0.05 ± 0.03 (0.093)	0.00 ± 0.02 (0.931)	**0.06 ± 0.02(0.016)**
	PC	−0.03 ± 0.04 (0.480)	−0.04 ± 0.02 (0.058)	0.01 ± 0.02 (0.566)
	PD	0.10 ± 0.05 (0.075)	−0.03 ± 0.02 (0.188)	−0.03 ± 0.02 (0.172)
	PH	0.01 ± 0.04 (0.740)	−0.01 ± 0.02 (0.652)	−0.03 ± 0.03 (0.299)
	PSD	0.08 ± 0.04 (0.070)	0.01 ± 0.02 (0.471)	−0.01 ± 0.02 (0.630)
Soil	SBD	−0.02 ± 0.03 (0.552)	0.02 ± 0.02 (0.131)	0.00 ± 0.03 (0.971)
	SL	**−0.07 ± 0.03 (0.035)**	0.03 ± 0.03 (0.216)	−0.00 ± 0.02 (0.836)
	SM	−0.06 ± 0.03 (0.053)	0.01 ± 0.02 (0.531)	−0.02 ± 0.02 (0.491)
	ST	----	0.01 ± 0.02 (0.499)	-0.05 ± 0.03(0.098)
Climate	Hum	**−0.09 ± 0.04 (0.048)**	**−0.07 ± 0.03 (0.006)**	0.02 ± 0.03 (0.419)
	Prec	**0.14 ± 0.04 (0.004)**	0.01 ± 0.02 (0.500)	−0.05 ± 0.03 (0.121)
	Temp	**−0.10 ± 0.05 (0.044)**	**−0.05 ± 0.02 (0.046)**	**−0.05 ± 0.02 (0.037)**
	Intercept	**0.86 ± 0.03 (<0.001)**	**0.63 ± 0.01 (<0.001)**	**0.59 ± 0.02 (<0.001)**

r^2 = adjusted coefficient of determination. Parameter estimated coefficients (± standard error) and *p*-values (in parentheses) are given for each predictor. Significant effects are in bold. Predictors abbreviations as in Table S1.

Table S6. Results of RE-ESF analysis between habitat characteristics and carabid diversity (inverse of Simpson dominance index) for the three grassland types.

Variables		Grassland Type		
		**Desert Steppe**	**Typical Steppe**	**Meadow Steppe**
**r^2**		0.31	0.19	0.25
Vegetation	PB	0.12 ± 0.10 (0.251)	0.01 ± 0.10 (0.882)	−0.17 ± 0.12(0.151)
	PC	0.06 ± 0.15 (0.669)	**0.22 ± 0.10 (0.032)**	−0.07 ± 0.11 (0.515)
	PD	−0.33 ± 0.19 (0.099)	0.16 ± 0.09 (0.075)	0.16 ± 0.11 (0.163)
	PH	−0.09 ± 0.15 (0.536)	0.02 ± 0.11 (0.878)	0.13 ± 0.15 (0.387)
	PSD	−0.31 ± 0.16 (0.061)	−0.04 ± 0.07 (0.558)	0.05 ± 0.11 (0.665)
Soil	SBD	0.01 ± 0.12 (0.919)	**−0.15 ± 0.07 (0.035)**	−0.09 ± 0.13 (0.491)
	SL	**0.27 ± 0.12 (0.027)**	−0.13 ± 0.11 (0.254)	0.06 ± 0.10 (0.566)
	SM	0.24 ± 0.12 (0.053)	−0.02 ± 0.07 (0.723)	0.10 ± 0.12 (0.421)
	ST	----	−0.12 ± 0.07 (0.110)	0.24 ± 0.16(0.130)
Climate	Hum	**0.41 ± 0.16 (0.017)**	**0.33 ± 0.11 (0.004)**	−0.11 ± 0.15 (0.448)
	Prec	**−0.51 ± 0.16 (0.004)**	−0.01 ± 0.08 (0.876)	0.16 ± 0.15 (0.264)
	Temp	**0.40 ± 0.18 (0.036)**	**0.23 ± 0.10 (0.019)**	**0.40 ± 0.12 (0.001)**
	Intercept	**1.43 ± 0.09 (<0.001)**	**2.25 ± 0.06 (<0.001)**	**2.50 ± 0.09 (<0.001)**

r^2 = adjusted coefficient of determination. Parameter estimated coefficients (± standard error) and *p*-values (in parentheses) are given for each predictor. Significant effects are in bold. Predictors abbreviations as in Table S1.

Table S7. Results of RE-ESF analysis between habitat characteristics and carabid diversity (inverse of Berger-Parker dominance index) for the three grassland types separately.

**Variables**		**Grassland Type**<br>**Desert Steppe**	**Typical Steppe**	**Meadow Steppe**
r^2		0.30	0.08	0.15
Vegetation	PB	0.09 ± 0.07 (0.210)	−0.05 ± 0.07 (0.526)	−0.13 ± 0.09(0.151)
	PC	0.05 ± 0.10 (0.610)	0.09 ± 0.07 (0.215)	−0.07 ± 0.08 (0.363)
	PD	−0.19 ± 0.14 (0.170)	0.05 ± 0.06 (0.361)	0.07 ± 0.08 (0.362)
	PH	−0.07 ± 0.11 (0.528)	0.01 ± 0.08 (0.945)	0.11 ± 0.11 (0.290)
	PSD	**−0.25 ± 0.11 (0.040)**	−0.01 ± 0.05 (0.799)	0.06 ± 0.08 (0.428)
Soil	SBD	0.04 ± 0.08 (0.593)	−0.09 ± 0.05 (0.070)	−0.06 ± 0.09 (0.549)
	SL	**0.17 ± 0.08 (0.051)**	−0.07 ± 0.08 (0.338)	0.01 ± 0.07 (0.917)
	SM	0.17 ± 0.08 (0.053)	0.00 ± 0.05 (0.991)	0.07 ± 0.09 (0.399)
	ST	----	−0.06 ± 0.05 (0.255)	0.15 ± 0.12(0.212)
Climate	Hum	**0.27 ± 0.12 (0.027)**	**0.17 ± 0.08 (0.038)**	−0.03 ± 0.11 (0.760)
	Prec	**−0.33 ± 0.11 (0.007)**	−0.03 ± 0.06 (0.574)	0.07 ± 0.11 (0.490)
	Temp	**0.27 ± 0.13 (0.050)**	0.09 ± 0.07 (0.210)	**0.19 ± 0.09 (0.027)**
	Intercept	**1.28 ± 0.06 (<0.001)**	**1.77 ± 0.04 (<0.001)**	**1.98 ± 0.06 (<0.001)**

r^2 = adjusted coefficient of determination. Parameter estimated coefficients (± standard error) and *p*-values (in parentheses) are given for each predictor. Significant effects are in bold. Predictors abbreviations as in Table S1.

Table S8. Results of Nested ANOVAs (with type of grasslands as fixed effect and sectors within types of grassland as random effect) followed by a Tukey tests for: Activity density, Chao 1, Margalef, Brillouin, Shannon, Simpson, Berger-Parker, Pielou, exponential Shannon-Wiener, inverse of Simpson dominance, and inverse of Berger-Parker dominance indices. r^2 = adjusted coefficient of determination.

	**Mean ± SE**			**Model Characteristics**		**Post hoc Tukey Test, Z-Value ± Std. Error (*p*-value)**		
	**Desert Steppe**	**Typical Steppe**	**Meadow Steppe**	**r^2**	**F-value (*p*-Value)**	**Desert Steppe—Meadow Steppe**	**Desert Steppe—Typical Steppe**	**Meadow Steppe—Typical Steppe**
Activity-density	8.244 ± 1.104	20.221± 1.707	15.511 ± 1.231	0.199	0.503 (0.648)	−0.342 ± 0.744 (0.888)	−0.514 ± 0.513 (0.571)	−0.1723 ± 0.705 (0.967)
Chao-1	1.854 ± 0.197	3.998 ± 0.169	4.184 ± 0.227	0.141	23.659 (0.015)	0.650 ± 0.096 (<0.001)	0.608 ± 0.124 (<0.001)	−0.043 ± 0.103 (0.907)
Margalef	0.368 ± 0.080	0.972 ± 0.039	1.098 ± 0.057	0.144	12.679 (0.034)	0.728 ± 0.210 (0.002)	0.602 ± 0.123 (<0.0001)	−0.126 ± 0.185 (0.766)
Brillouin	0.219 ± 0.051	0.648 ± 0.027	0.651 ± 0.033	0.145	21.364 (0.017)	0.432 ±0.067 (<0.001)	0.423 ±0.095 (<0.001)	−0.009 ±0.082 (0.994)
Shannon-Wiener	0.308 ± 0.069	0.850 ± 0.032	0.908 ± 0.043	0.143	19.680 (0.019)	0.599 ± 0.097 (<0.001)	0.536 ± 0.109 (<0.001)	−0.063 ± 0.085 (0.738)
Simpson	0.821 ± 0.039	0.531 ± 0.016	0.499 ± 0.021	0.136	7.512 (0.068)	−0.545 ± 0.146 (0.001)	−0.458 ± 130 (0.001)	0.087 ± 0.097 (0.6369)
Berger-Parker	0.861 ± 0.033	0.633 ± 0.014	0.590 ± 0.020	0.126	10.410 (0.045)	−0.415 ± 0.115 (<0.001)	−0.321 ± 0.075(<0.001)	0.094 ± 0.097 (0.590)
Pielou	0.307 ± 0.062	0.681 ± 0.021	0.728 ± 0.028	0.133	25.665 (0.013)	0.420 ± 0.066 (<0.001)	0.373 ± 0.055 (<0.001)	−0.047 ± 0.049 (0.597)
exponential Shannon-Wiener	1.523 ± 0.138	2.590 ± 0.078	2.782 ± 0.113	0.116	12.930 (0.034)	0.391 ± 0.078 (<0.001)	0.344 ± 0.083 (<0.001)	−0.047 ± 0.062 (0.732)
inverse of Simpson dominance	1.426 ± 0.119	2.246 ± 0.064	2.502 ± 0.100	0.114	7.505 (0.068)	0.546 ± 0.146 (0.001)	0.459 ± 0.130 (0.001)	−0.087 ± 0.097 (0.637)
inverse of Berger-Parker dominance	1.279 ± 0.082	1.766 ± 0.043	1.980 ± 0.068	0.095	10.410(0.046)	0.415± 0.115 (<0.001)	0.321 ± 0.075 (<0.001)	−0.094± 0.097 (0.590)
